# Increased risk for chronic comorbid disorders in patients with inflammatory arthritis: a population based study

**DOI:** 10.1186/1471-2296-14-199

**Published:** 2013-12-23

**Authors:** Jennie Ursum, Mark MJ Nielen, Jos WR Twisk, Mike JL Peters, François G Schellevis, Michael T Nurmohamed, Joke C Korevaar

**Affiliations:** 1 NIVEL (Netherlands Institute for Health Services Research), PO Box 1568, Utrecht 3500, BN, the Netherlands; 2 EMGO + Institute for Health and Care Research, VU University, Amsterdam, Netherlands; 3 Department of Epidemiology and Biostatistics, VU University Medical Centre, Amsterdam, Netherlands; 4 Department of Internal Medicine, VU University Medical Centre, Amsterdam, Netherlands; 5 Department of General Practice/EMGO Institute for Health and Care Research, VU University Medical Centre, Amsterdam, the Netherlands; 6 Department of Rheumatology, VU University Medical Centre, Amsterdam, Netherlands; 7 Jan van Breemen Research Institute | Reade, Amsterdam, the Netherlands

**Keywords:** Inflammatory arthritis, Comorbidity, General practice, Disease onset, Chronic disease

## Abstract

**Background:**

Studies determining the development of a wide variety of different comorbid disorders in inflammatory arthritis (IA) patients are scarce, however, this knowledge could be helpful in optimising preventive care in IA patients. The aim of this study is to establish the risk that new chronic comorbid disorders in newly diagnosed patients with IA in a primary care setting are developed.

**Methods:**

This is a nested-case–control study from 2001–2010 using data from electronic medical patient records in general practice. In total, 3,354 patients with newly diagnosed IA were selected. Each patient was matched with two control patients of the same age and sex in the same general practice. The development of 121 chronic comorbid disorders of index and control patients was compared using Cox regression.

**Results:**

After a median follow-up period of 2.8 years, 56% of the IA-patients had developed at least one chronic comorbid disorder after the onset of IA, compared to 46% of the control patients (p < 0.05). The most frequent developed comorbid disorders after the onset of IA were of cardiovascular (23%), and musculoskeletal (17%) origin. The highest hazard ratios (HRs) were found for anaemia (HR 2.0 [95% CI: 1.4-2.7]) osteoporosis (HR 1.9 [1.4-2.4]), and COPD (HR 1.8 [1.4-2.3]).

**Conclusion:**

Patients with IA developed more chronic comorbid disorders after the onset of IA than one might expect based on age and sex. Since comorbidity has a large impact on the disease course, quality of life, and possibly on treatment itself, prevention of comorbidity should be one of the main targets in the treatment of IA patients.

## Background

Inflammatory arthritis (IA) is defined as a group of chronic rheumatic diseases, including rheumatoid arthritis (RA), psoriatic arthritis (PsA) and ankylosing spondylitis (AS). These are all auto-immune diseases characterized by inflammation of joints and structural changes of bone and cartilage, with similar treatments. The prevalence of IA in general practice was 4.8 per 1000 patients in the Netherlands in 2011. In an average Dutch general practice around 2350 patients are listed, so approximately 12 patients with IA are listed in a general practice.

Like many patients with a chronic disease, the majority of patients with IA have at least one other chronic disorder, the observed percentages ranged from 16 to 34%[1-5]. Several chronic diseases such as cardiovascular diseases [6,7] pulmonary diseases [8] and gastrointestinal diseases [9] are known to be increased present in IA patients [10-12]. The impact of comorbidity is high, as more chronic diseases result in more disability and diminished health related quality of life [13]. Additionally, (multiple) comorbid conditions in IA patients often lead to worse clinical outcomes [14].

Longitudinal studies in community-based settings establishing the development of a wide variety of different comorbid disorders in IA patients are scarce [15,16]. Up to now most studies addressing IA patients focus either on the development of one specific comorbid disorder or one group of disorders. Moreover, limited attention is paid to the question whether the observed frequency of newly developed chronic disorders is higher than one might expect based on age and sex, since a suitable control group is often lacking.

A few studies have indicated that the increased risk of developing new chronic diseases in IA patients might be due to a possible lack of optimal preventive care [17-19]. Therefore, it could be helpful for optimising preventive care in IA patients to know the increased risk for a broad variety of comorbid diseases.

This study aims to establish the risk that new chronic comorbid disorders in newly diagnosed patients with IA in a primary care setting are developed.

## Methods

### Study population

The Dutch Primary Care Database [20] is a network of, on average, 83 general practices with about 335,000 registered patients and it was started in 1994. General Practitioners (GPs) record medical information from patients routinely in electronic medical records (EMR), using the International Classification of Primary Care – version 1 (ICPC). The ICPC is a classification method for primary care encounters, accepted by the WHO [21,22]. EMRs contain information on consultations, morbidity, prescriptions, and referrals to other healthcare professionals. We have used data from the period 2001–2010. Available data are representative for the whole Dutch population [20].

### Selection of IA patients and controls

We have selected all newly diagnosed patients with inflammatory arthritis (IA) aged >30 years, based on ICPC-code L88 - Rheumatoid arthritis and related disorders [21,22]. A selection purely based on this code could contain some non-IA patients, so we applied a well-established algorithm to exclude non-IA patients [23]. Based on this algorithm patients were included if they had a combination of specific anti-rheumatic drug prescriptions, or if they had more than one visit to their GP with code L88, or were 60 years or younger. To ensure that only newly diagnosed IA patients were included, we excluded patients with an L88-code within 365 days from start of registration. This was based on the assumption that all IA patients will go to their GP at least once a year for IA-related complaints.

To determine whether IA patients develop more chronic diseases than might be expected based on age and sex, we matched all IA-patients (index patients) with two control patients (non-IA patients, i.e. patients without an ICPC code L88) from the same general practice. The controls were matched regarding age (timeframe of 5 years) and sex. The date of diagnosis of IA was taken as the date of inclusion of the matched control. Both index and control patients were only included if they had at least 90 days of follow-up.

### Chronic diseases and diagnostic clusters

The onset of one hundred and twenty-one chronic diseases was determined based on a study of O’Halloran *et al.*, Additional file 1[24]. All these ICPC codes were categorised within 17 ICPC chapters, such as cardiovascular, respiratory or neurological diseases (further called ‘main disease groups’). Additionally, separate ICPC codes do not always give a complete picture of the disease, therefore, certain ICPC codes were combined into diagnostic clusters, for example uncomplicated hypertension (K86) and complicated hypertension (K87) were combined. These clusters were based on the selection of chronic diseases of the National Institute of Public Health and the Environment from the Netherlands [25] and the Second Dutch National Survey of General Practice [26]. An overview of the fifteen used diagnostic clusters is presented in Additional file 2.

### Statistical analysis

Only chronic comorbid disorders with a prevalence of at least 2% are presented. Gout (T92), psoriasis (S91) and osteoarthritis (OA) (diagnostic cluster) were excluded from the analysis, because these diseases may be interrelated to IA. All chronic comorbid disorders diagnosed after inclusion were indicated as newly developed. The development of new chronic diseases was tested in IA patients and control patients with a Chi-square test. Thereafter, we applied multivariable Cox regression analyses, adjusting for age, sex and number of prevalent chronic diseases at baseline, to determine the difference between IA and control patients. We performed sensitivity analyses, limiting the multivariable Cox regression analysis to 1) cases and matched controls with at least one year of follow-up, and 2) including only new chronic comorbid disorders diagnosed 90 days after inclusion. The latter restriction was chosen because the exact date of onset of chronic diseases is not always clear. A diagnosis made 90 days or more after the diagnosis IA is very likely to be an onset after the onset of IA. Finally, logistic regression was performed, by filling in the regression equation, and the absolute change for developing a chronic disease was calculated.

All statistical analyses were performed with Stata/SE 12.1 (StataCorp, College Station, TX, USA).

### Ethical approval

The study was carried out according to Dutch legislation on privacy. The privacy regulation of the study was registered at the Dutch Data Protection Authority. According to Dutch legislation, nor obtaining informed consent nor approval by a medical ethics committee was obligatory for observational studies.

## Results

We identified 3,356 patients with IA and matched 6,708 controls. The baseline characteristics are shown in Table 1. Nearly two-thirds of the patients were female (64%), the mean age was 55 years (SD = 15) and median follow-up duration was 2.8 years.

**Table 1:** Baseline characteristics

	**IA patients**	**Matched controls**
N (ratio 1:2)	3,354	6,708
Sex		
Female	63.7%	63.7%
Age, years		
Mean (sd)	55 (15)	55 (15)
Follow-up, years		
Median (iqr)	2.8 (1.7-4.9)	2.7 (1.6-4.9)
Number of chronic diseases at inclusion*		
Median (iqr)	1 (0–3)	1 (0–2)
Year of inclusion,		
2001	<1%	<1%
2002	16%	16%
2003	12%	12%
2004	9%	9%
2005	10%	10%
2006	10%	10%
2007	13%	13%
2008	11%	11%
2009	11%	11%
2010	9%	9%

IA = inflammatory arthritis sd= standard deviation, iqr=interquartile range, min= minimum, max=maximum.

*without IA.

**Figure 1:**
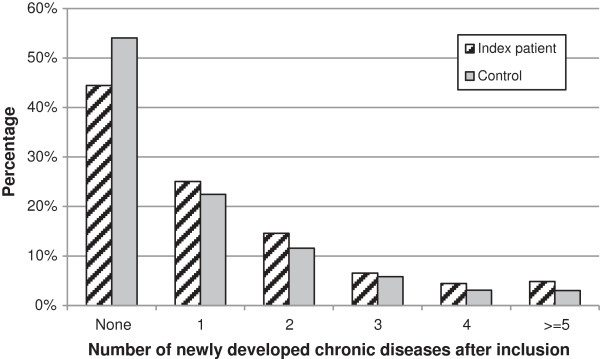
Percentage of patients who developed new chronic diseases after inclusion.

The number of new chronic comorbid disorders differed significantly between IA and control patients (p = 0.001, Figure 1). In total, 56% of the IA patients developed one or more chronic comorbid disorder after the diagnosis of IA, compared to 46% of the control patients (Figure 1). One quarter of the IA patients developed one chronic comorbid disorder and 31% developed two or more chronic comorbidities. Five per cent of the IA patients developed five or more chronic comorbid disorders during follow-up.

Table 2 shows only chronic diseases with a prevalence of at least 2% in IA patients or controls. In total, 23% of the IA patients and 18% of the control patients developed a cardiovascular disease (HR: 1.3 (1.2 – 1.5) (Table 2). In total, 17% of the IA patients and 14% of the control patients (HR: 1.3, 95% CI: 1.1 – 1.4) developed musculoskeletal diseases. The frequency of the remaining disease groups varied between 3 and 10% for IA patients and 1 to 9% for control patients.

**Table 2:** Percentage of IA patients and control patients with new chronic comorbid disorders after diagnosis†

	**IA patients**	**Matched controls**	**HR crude**	**95% CI**	**HR adjusted**^**#**^	**95% CI**	**p-value**
Main disease group*							
Diagnosis							
**Blood and blood forming organs**	**3%**	**1%**	**2.0**	**(1.5-2.6)**	**1.9**	**(1.4-2.6)**	**<0.001**
Anaemia^§^	2%	1%	2.0	(1.4-2.7)	2.0	(1.4-2.7)	<0.001
**Respiratory**	**7%**	**5%**	**1.6**	**(1.3-1.9)**	**1.5**	**(1.3-1.8)**	**<0.001**
COPD^§^	4%	2%	1.8	(1.4-2.3)	1.8	(1.4-2.3)	<0.001
Asthma (R96)	4%	3%	1.4	(1.1-1.8)	1.3	(1.0-1.7)	0.015
**Neurological**	**8%**	**5%**	**1.5**	**(1.3-1.7)**	**1.4**	**(1.2-1.6)**	**<0.001**
Carpal tunnel syndrome (N93)	3%	2%	1.8	(1.4-2.4)	1.7	(1.3-2.3)	<0.001
Peripheral neuritis/neuropathy (N94)	3%	2%	1.9	(1.5-2.5)	1.7	(1.3-2.2)	<0.001
**Psychological**	**9%**	**7%**	**1.3**	**(1.1-1.5)**	**1.3**	**(1.1-1.4)**	**0.003**
Anxiety disorder^§^	2%	1%	1.5	(1.1-2.1)	1.4	(1.0-2.0)	0.035
Depressive disorder (p76)	4%	3%	1.4	(1.1-1.8)	1.4	(1.1-1.7)	0.012
**Cardiovascular**	**23%**	**18%**	**1.3**	**(1.2-1.4)**	**1.3**	**(1.2-1.5)**	**<0.001**
Phlebitis/thrombophlebitis (K94)	2%	1%	1.8	(1.3-2.4)	1.7	(1.2-2.3)	0.002
Varicose veins of leg (K95)	3%	2%	1.4	(1.1-1.8)	1.4	(1.1-1.8)	0.009
Hypertension^§^	12%	9%	1.3	(1.1-1.5)	1.3	(1.1-1.5)	0.001
Heart failure (K77)	3%	2%	1.4	(1.1-1.9)	1.5	(1.1-2.0)	0.005
**Digestive**	**5%**	**4%**	**1.3**	**(1.1-1.6)**	**1.3**	**(1.0-1.5)**	**0.02**
**Musculoskeletal**^**b**^	**17%**	**14%**	**1.3**	**(1.1-1.4)**	**1.3**	**(1.1-1.4)**	**<0.001**
Osteoporosis (L95)	3%	2%	1.9	(1.4-2.4)	1.9	(1.5-2.5)	<0.001
Spinal Cord^§^	9%	7%	1.4	(1.2-1.6)	1.3	(1.1-1.5)	0.001
**Endocrine**^**a**^	**10%**	**9%**	**1.1**	**(0.9-1.2)**	**1.0**	**(0.9-1.2)**	**0.514**
**Other**							
Vertiginous syndrome (H82)	3%	2%	1.5	(1.2-1.9)	1.4	(1.1-1.8)	0.005
Cancer^§^	5%	4%	1.3	(1.1-1.6)	1.3	(1.1-1.6)	0.004
Eczema^§^	5%	4%	1.3	(1.0-1.6)	1.3	(1.0-1.6)	0.023

†Patients or controls may have several chronic comorbid disorders.

*Diagnoses included are listed in supplementary file. The ICPC codes within the cluster Cancer were excluded from the main groups.

HR = odds ratio, CI = confidence interval.

#Adjusted for age and sex.

§Cluster.

^a^Gout excluded.

^b^Cluster osteoarthritis excluded.

The highest risks to develop individual diseases for IA patients compared to control patients were found for anaemia (HR: 2.0), osteoporosis (HR: 1.9), COPD (HR: 1.8), carpal tunnel syndrome (HR: 1.7), and peripheral neuritis (HR 1.7).

The sensitivity analyses did not alter our findings, just all effects were slightly reduced due to a loss of power.

**Figure 2:**
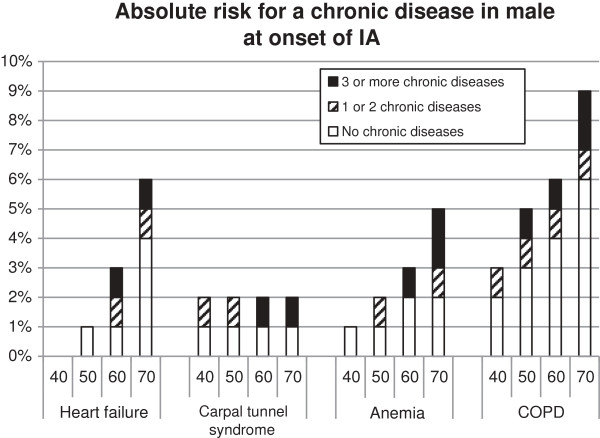
**The absolute risk on a chronic disease accumulated for the categories no. 1 or 2 and 3 or more diseases.** When no bar is presented, the risk for developing a new chronic disease is 0%. Absence of the other two categories means no additional risk with respect to the previous category. For example: a 60-year old male with no additional chronic disease has 2% risk to develop anemia. A 60-year old male with one or two chronic diseases at IA onset has also 2% risk to develop anemia -no additional bar for one or two chronic diseases is presented-. A 60-year old male with three or more chronic diseases at IA onset has 3% risk to develop anemia.

**Figure 3:**
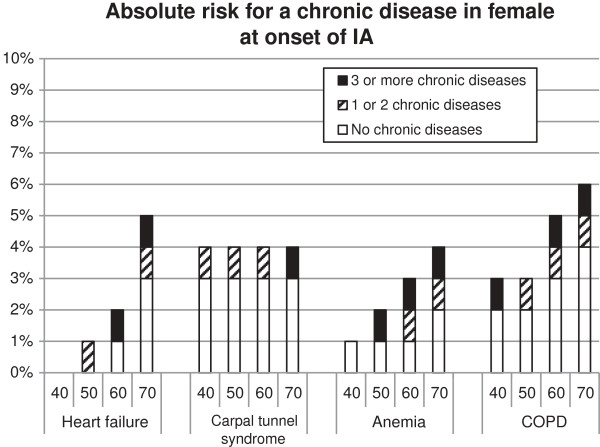
**The absolute risk of developing a new chronic disease stacked for the categories no. 1 or 2, and 3 or more diseases.** When no bar is presented, the risk for developing a new chronic disease is 0%. Absence of a category means no additional risk with respect to de previous category. For example: a 70-year old female with no additional chronic disease at IA onset has 4% risk to develop COPD. A 70-year old female with one or two chronic diseases at IA onset has 5% risk to develop COPD. A 70-year old female with three or more chronic diseases at IA onset has 6% risk to develop COPD.

So what do these results mean for a specific patient in the general practice? To provide deeper insight into the risk for a specific patient, the changes to develop COPD, carpal tunnel syndrome, anaemia or heart failure were calculated, see Figure 2 and 3. Women have a higher risk to develop carpal tunnel syndrome compared to men, for the other diseases it is the other way around. The presence and the number of chronic diseases result in a higher risk to develop another chronic disease. Additionally, higher age resulted in a higher risk to develop an additional chronic disease. For example: A male of 60 years at the onset of IA without additional chronic disease has a risk of 4% to develop COPD within 2.8 yrs. A male aged 60 and with one or two chronic diseases at IA onset has a risk of 5% risk to develop COPD within 2.8 yrs. A male aged 60 and with three or more chronic diseases at IA onset has 6% risk to develop COPD within this time frame.

## Discussion

### Summary

Patients develop more chronic comorbid disorders after the onset of IA than expected based on their age and sex. In a median follow-up period of 2.8 years, 56% of the IA patients have developed at least one chronic disease compared to 46% in the matched control group. Of the main disease groups, cardiovascular diseases were the most common newly developed comorbidities, whereas the highest risk for individual diseases was found for anaemia, osteoporosis and COPD.

### Comparison with existing literature

A study by Kapetanovic *et al.* found that 82% of the RA patients developed a chronic comorbid disease after disease onset [27]. This percentage is higher compared to our study (56%), but the follow-up period was much longer, up to twenty years, and they used self-reported comorbidities instead of recorded in the EMR of the GP.

Several recent studies demonstrated that patients with IA have an increased risk of cardiovascular disease.^10;11^ The result of our study supports this finding. However, we did not find an increased risk for some common reported cardiovascular diseases in patients with IA, like ischaemic heart disease, atrial fibrillation flutter or stroke [10-12]. These diseases might develop after a longer period than our median follow-up duration of nearly three years. We found a small but increased risk for cancer in IA patients; this finding is supported by a meta-analysis of Smitten *et al*. who found increased risks for several cancer types in IA patients [28].

We found an increased risk that IA patients develop COPD. This is in line with another study [29]. Patients with COPD have partly the same clinical and pathophysiological characteristics as patients with IA, like inflammation. Moreover, the inflammatory response is self-perpetuating and exacerbations occur in both IA and COPD [30]. Finally, both diseases share risk factors like smoking and physical inactivity. Apart from COPD, inflammation could also play a role in the development of osteoporosis, anaemia, carpal tunnel syndrome and cardiovascular diseases [31-33]. For all these diseases an increased risk was found in the current study. Malnutrition caused by inflammation and hormonal factors may subsequently play a role in the development of osteoporosis as well as the use of glucocorticoids leading to glucocorticoid-induced osteoporosis [34-36]. Carpal tunnel syndrome might be the result of IA related inflammation as well, as inflammation of the synovial tissue of the flexor tendons can also cause increased pressure in the carpal tunnel [33].

In our study, over half of all IA patients developed one or more new diseases within a median follow-up period of nearly 3 years. Furthermore, the newly developed diseases represent a broad spectrum of diseases, so no fixed sequence or combinations of new diseases was observed. This is confirmed in studies among patients with multimorbidity, as patients with a chronic disease often have more than one chronic disease, as multimorbidity occurs in more than two-thirds of the elderly with common chronic diseases [37]. Additionally, another study found that the cumulative incidence rates of one or more new morbidities rapidly increase with the number of morbidities already present [38,39]. The sequence of developing additional chronic comorbid disorders may be influenced by different factors like medication use, the combination of disease already acquired, or a common underlying mechanism [40].

### Strengths and limitations

A strength of this study is that all Dutch citizens are listed within a general practice, and the GP is the first professional to be consulted for health problems. Therefore, the GP has a complete view on the patients’ lifetime health status.^37^ Moreover, Dutch GPs obtain information of diagnosis made by the medical specialist, since the Dutch guidelines for optimising GP-medical specialist communication support medical specialists to inform GPs about the referred patient. Beside these advantages, this study has some limitations. First, we have excluded gout, osteoarthritis and psoriasis from the results, because these diseases are too much interrelated with IA. Therefore, it is difficult to conclude whether it is an isolated disease or part of IA. Patients with a combination of one of these diseases and an IA suspicion (L88 code registered) could have been misclassified. Second, we have used the ICPC code L88 to identify possible IA patients and we have used an algorithm to distinguish IA from non-IA. However, it was not possible to exclude all non-IA patients, this could have resulted in an underestimation of the true hazard ratios. Furthermore, the follow-up of three years may be too short a period to develop some chronic comorbid disorders, like e.g. ischaemic heart disease.

### Implications for research and/or practice

More than half of the patients with IA developed one or more chronic diseases after the onset of IA. As inflammatory processes are a common risk factor for several other comorbidities, tight control of inflammation seems of great importance. Also, attention for other common or specific risk factors, like smoking for COPD, or glucocorticoid use for osteoporosis, can improve the primary or secondary preventive care for IA patients and may prevent or postpone the occurrence of some comorbidities. The attention for the development of chronic diseases in older patients should be intensified and also the presence of chronic diseases should be taken into account.

## Conclusion

Patients with IA develop more chronic comorbid disorders after diagnosis of IA than is to be expected based on age and sex. The most frequent chronic comorbidities obtained after diagnose can be categorised in cardiovascular, musculoskeletal and neurological diseases. Since comorbidity has an impact on the disease course, on quality of life, and possibly on the treatment itself, preventive care should become a more explicit treatment target.

## Competing interests

The authors declare that they have no competing interests.

## Authors’ contributions

JU performed the study according to protocol, analyzed the data and wrote the manuscript. MMJN, JWRT, MJLP and JCK helped writing the manuscript and contributed substantially to the statistical analysis and interpretation of the data. JCK, FGS and MTN supervised the study and developed the study protocol. All authors read and approved the final manuscript.

## Pre-publication history

The pre-publication history for this paper can be accessed here:

http://www.biomedcentral.com/1471-2296/14/199/prepub

## Supplementary Material

Additional file 1: Table S1 ICPC codes of chronic diseases. Click here for file

Additional file 2: Table S2 Overview of ICPC codes that were combined into disease clusters. Click here for file
