# Supplementary material for: Increased risk for chronic comorbid disorders in patients with inflammatory arthritis: a population based study
Source: BMC Fam Pract. 2013 Dec 23;14:199. doi: 10.1186/1471-2296-14-199 (PMC3909051; doi:10.1186/1471-2296-14-199)
Supplement: Additional file 1: Table S1 — ICPC codes of chronic diseases. [file 1471-2296-14-199-S1.docx]

**Appendix I. ICPC codes of chronic diseases**

| **Main disease group*** | **ICPC code** | **Diagnosis** | **Diagnostic cluster**  **(see also Appendix II)** |
| --- | --- | --- | --- |
|  | A70 | Tuberculosis |  |
|  | A79 | Malignancy NOS | Cancer |
|  | A90 | Congenital anomaly OS/multiple | Congenital diseases |
|  | B72 | Hodgkin's disease/lymphoma | Cancer |
|  | B73 | Leukaemia | Cancer |
|  | B74 | Malignant neoplasm blood other | Cancer |
| Blood | B78 | Hereditary haemolytic anaemia |  |
| Blood | B81 | Anaemia, Vitamin B12/folate deficiency | Anaemia |
| Blood | B82 | Anaemia other/unspecified | Anaemia |
| Blood | B83 | Purpura coagulation defects |  |
| Blood | B90 | HIV infection AIDS |  |
|  | D74 | Malignant neoplasm stomach | Cancer |
|  | D75 | Malignant neoplasm colon/rectum | Cancer |
|  | D76 | Malignant neoplasm pancreas | Cancer |
|  | D77 | Malig. neoplasm digest other/NOS | Cancer |
|  | D84 | Oesophagus disease | Congenital diseases |
| Digestive | D85 | Duodenal ulcer | Ulcer |
| Digestive | D86 | Peptic ulcer other | Ulcer |
| Digestive | D92 | Diverticular disease |  |
| Digestive | D93 | Irritable bowel syndrome |  |
| Digestive | D94 | Chronic enteritis ulcerative colitis |  |
| Digestive | D97 | Cirrhosis Liver disease NOS |  |
| Digestive | D98 | Cholecystitis cholelithiasis |  |
| Eye | F83 | Retinopathy | Visual impairment |
| Eye | F84 | Macular degeneration | Visual impairment |
| Eye | F92 | Cataract | Visual impairment |
| Eye | F93 | Glaucoma | Visual impairment |
|  | F94 | Blindness | Visual impairment |
| Ear | H82 | Vertiginous syndrome |  |
| Ear | H84 | Presbyacusis | Hearing impairment |
| Ear | H85 | Acoustic trauma | Hearing impairment |
| Ear | H86 | Deafness | Hearing impairment |
|  | K73 | Congenital anomaly cardiovascular | Congenital diseases |
| Cardiovascular | K74 | Ischemic heart disease with angina | Ischaemic heart disease |
| Cardiovascular | K75 | Acute myocardial infarction | Ischaemic heart disease |
| Cardiovascular | K76 | Ischemic heart disease without angina | Ischaemic heart disease |
| Cardiovascular | K77 | Heart failure |  |
| Cardiovascular | K78 | Atrial fibrillation flutter |  |
| Cardiovascular | K79 | Paroxysmal tachycardia |  |
| Cardiovascular | K80 | Cardiac arrhythmia NOS |  |
| Cardiovascular | K81 | Heart arterial murmur NOS |  |
| Cardiovascular | K82 | Pulmonary heart disease |  |
| Cardiovascular | K83 | Heart valve disease NOS |  |
| Cardiovascular | K84 | Heart disease other |  |
| Cardiovascular | K86 | Hypertension uncomplicated | Hypertension |
| Cardiovascular | K87 | Hypertension complicated | Hypertension |
| Cardiovascular | K88 | Postural hypotension |  |
| Cardiovascular | K89 | Transient cerebral ischaemia | Stroke |
| Cardiovascular | K90 | Stroke/ cerebrovascular accident | Stroke |
| Cardiovascular | K91 | Atherosclerosis |  |
| Cardiovascular | K92 | Peripheral vascular disease |  |
| Cardiovascular | K93 | Pulmonary embolism |  |
| Cardiovascular | K94 | Phlebitis thrombophlebitis |  |
| Cardiovascular | K95 | Varicose veins of leg |  |
|  | L71 | Malignant neoplasm musculoskeletal | Cancer |
| Musculoskeletal | L83 | Neck syndrome | Spinal cord |
| Musculoskeletal | L84 | Back syndrome w/o radiating pain | Spinal cord |
| Musculoskeletal | L85 | Acquired deformity of spine | Spinal cord |
| Musculoskeletal | L86 | Back syndrome with radiating pain | Spinal cord |
|  | L88 | Rheumatoid arthritis and related diseases | (cases) |
|  | L89 | Osteoarthrosis of hip | Osteoarthritis |
|  | L90 | Osteoarthrosis of knee | Osteoarthritis |
|  | L91 | Osteoarthrosis other | Osteoarthritis |
| Musculoskeletal | L92 | Shoulder syndrome |  |
| Musculoskeletal | L93 | Tennis elbow |  |
| Musculoskeletal | L95 | Osteoporosis |  |
| Neurological | N73 | Neurological infection other |  |
|  | N74 | Malignant neoplasm nervous system | Cancer |
| Neurological | N75 | Benign neoplasm nervous system |  |
|  | N76 | Neoplasm nervous system unspecified | Congenital diseases |
|  | N85 | Congenital anomaly neurological | Congenital diseases |
| Neurological | N86 | Multiple sclerosis |  |
| Neurological | N87 | Parkinsonism |  |
| Neurological | N88 | Epilepsy |  |
| Neurological | N89 | Migraine | Migraine |
| Neurological | N90 | Cluster headache | Migraine |
| Neurological | N92 | Trigeminal neuralgia | Migraine |
| Neurological | N93 | Carpal tunnel syndrome |  |
| Neurological | N94 | Peripheral neuritis neuropathy |  |
| Psychological | P15 | Chronic alcohol abuse |  |
| Psychological | P70 | Dementia |  |
| Psychological | P71 | Organic psychosis other |  |
| Psychological | P72 | Schizophrenia |  |
| Psychological | P73 | Affective psychosis |  |
| Psychological | P74 | Anxiety disorder/anxiety state | Anxiety disorders |
| Psychological | P75 | Somatisation disorder |  |
| Psychological | P76 | Depressive disorder |  |
| Psychological | P78 | Neuraesthenia surmenage |  |
| Psychological | P79 | Phobia/compulsive disorder | Anxiety disorders |
| Psychological | P80 | Personality disorder |  |
| Psychological | P85 | Mental retardation |  |
| Psychological | P98 | Psychosis NOS other |  |
|  | R84 | Malignant neoplasm bronchus/lung | Cancer |
|  | R85 | Malinant neoplasm respiratory, other | Cancer |
| Respiratory | R90 | Hypertrophy tonsils adenoids |  |
| Respiratory | R91 | Chronic bronchitis | COPD |
| Respiratory | R95 | Emphysema/chronic obstructive pulmonary disease | COPD |
| Respiratory | R96 | Asthma |  |
|  | S77 | Malignant neoplasm of skin | Cancer |
| Skin | S86 | Dermatitis seborrhoeic | Eczema |
| Skin | S87 | Dermatitis/atopic eczema | Eczema |
|  | S91 | Psoriasis |  |
| Endocrine | T06 | Anorexia nervosa bulimia |  |
|  | T71 | Malignant neoplasm thyroid | Cancer |
| Endocrine | T81 | Goitre |  |
| Endocrine | T85 | Hyperthyroidism thyrotoxicosis |  |
| Endocrine | T86 | Hypothyroidism myxoedema |  |
| Endocrine | T90 | Diabetes |  |
|  | T92 | Gout |  |
| Endocrine | T93 | Lipid disorder |  |
|  | U75 | Malignant neoplasm of kidney | Cancer |
|  | U76 | Malignant neoplasm of kidney | Cancer |
|  | U77 | Malignant neoplasm urinary other | Cancer |
|  | U88 | Glomerulonephritis nephrosis |  |
|  | W15 | Infertility/ subfertility |  |
|  | W72 | Malignant neoplasm relate to pregnancy | Cancer |
|  | X74 | Pelvic inflammatory disease |  |
|  | X75 | Malignant neoplasm cervix | Cancer |
|  | X76 | Malignant neoplasm breast female | Cancer |
|  | X77 | Malignant neoplasm genital other (f) | Cancer |
| Male Genital | Y78 | Fibromyoma uterus | Cancer |
| Male Genital | Y85 | Benign prostatic hypertrophy |  |
| NOS: not otherwise specified  * Diagnosis within the cluster Cancer not included in the main disease groups | | | |
