# Supplementary material for: Increased risk for chronic comorbid disorders in patients with inflammatory arthritis: a population based study
Source: BMC Fam Pract. 2013 Dec 23;14:199. doi: 10.1186/1471-2296-14-199 (PMC3909051; doi:10.1186/1471-2296-14-199)
Supplement: Additional file 2: Table S2 — Overview of ICPC codes that were combined into disease clusters. [file 1471-2296-14-199-S2.docx]

**Appendix II. Overview of ICPC codes that were combined into disease clusters**

| **Disease Clusters** | **ICPC codes** |
| --- | --- |
| Anaemia | B81 B82 |
| Cancer | A79 B72 B73 D74 D75 D76 D77 L71 N74 R84 R85 S77 T71 U75 U76 U77 W72 X75 X76 X77 Y77 Y78 B74 |
| Congenital diseases | A90 D84 K73 N76 N85 |
| Ulcer | D85 D86 |
| Visual impairment | F83 F84 F92 F93 F94 |
| Hearing impairment | H84 H85 H86 |
| Ischaemic hart disease | K74 K75 K76 |
| Hypertension | K86 K87 |
| Stroke | K89 K90 |
| Spinal cord | L83 L84 L85 L86 |
| Osteoarthritis | L89 L90 L91 |
| Migraine | N89 N90 N92 |
| Anxiety disorders | P74 P79 |
| COPD | R91 R95 |
| Eczema | S87 S86 |
